# Integrative multiomic analysis identifies genes associated with cuticular wax biogenesis in adult maize leaves

**DOI:** 10.1093/g3journal/jkae241

**Published:** 2024-10-10

**Authors:** Meng Lin, Harel Bacher, Richard Bourgault, Pengfei Qiao, Susanne Matschi, Miguel F Vasquez, Marc Mohammadi, Sarah van Boerdonk, Michael J Scanlon, Laurie G Smith, Isabel Molina, Michael A Gore

**Affiliations:** Plant Breeding and Genetics Section, School of Integrative Plant Science, Cornell University, Ithaca, NY 14853, USA; Plant Breeding and Genetics Section, School of Integrative Plant Science, Cornell University, Ithaca, NY 14853, USA; Department of Biology, Algoma University, Sault Ste. Marie, ON P6A 2G4, Canada; Plant Biology Section, School of Integrative Plant Science, Cornell University, Ithaca, NY 14853, USA; Department of Cell and Developmental Biology, University of California San Diego, La Jolla, CA 92093, USA; Department of Cell and Developmental Biology, University of California San Diego, La Jolla, CA 92093, USA; Department of Biology, Algoma University, Sault Ste. Marie, ON P6A 2G4, Canada; Department of Biology, Algoma University, Sault Ste. Marie, ON P6A 2G4, Canada; Plant Biology Section, School of Integrative Plant Science, Cornell University, Ithaca, NY 14853, USA; Department of Cell and Developmental Biology, University of California San Diego, La Jolla, CA 92093, USA; Department of Biology, Algoma University, Sault Ste. Marie, ON P6A 2G4, Canada; Plant Breeding and Genetics Section, School of Integrative Plant Science, Cornell University, Ithaca, NY 14853, USA

**Keywords:** maize, leaf cuticular waxes, leaf cuticular conductance, natural variation, genome-wide association study, transcriptome-wide association study, Plant Genetics and Genomics

## Abstract

Studying the genetic basis of leaf wax composition and its correlation with leaf cuticular conductance (*g*_c_) is crucial for improving crop productivity. The leaf cuticle, which comprises a cutin matrix and various waxes, functions as an extracellular hydrophobic layer, protecting against water loss upon stomatal closure. To address the limited understanding of genes associated with the natural variation of adult leaf cuticular waxes and their connection to *g*_c_, we conducted statistical genetic analyses using leaf transcriptomic, metabolomic, and physiological data sets collected from a maize (*Zea mays* L.) panel of ∼300 inbred lines. Through a random forest analysis with 60 cuticular wax traits, it was shown that high molecular weight wax esters play an important role in predicting *g*_c_. Integrating results from genome-wide and transcriptome-wide association studies via a Fisher's combined test revealed 231 candidate genes detected by all 3 association tests. Among these, 11 genes exhibit known or predicted roles in cuticle-related processes. Throughout the genome, multiple hotspots consisting of genome-wide association study signals for several traits from 1 or more wax classes were discovered, identifying 4 additional plausible candidate genes and providing insights into the genetic basis of correlated wax traits. Establishing a partially shared genetic architecture, we identified 35 genes for both *g*_c_ and at least 1 wax trait, with 4 considered plausible candidates. Our study enhances the understanding of how adult leaf cuticle wax composition relates to *g*_c_ and implicates both known and novel candidate genes as potential targets for optimizing productivity in maize.

## Introduction

The cuticle covers the surface of aerial organs of all land plants. Being predominantly hydrophobic, cuticles provide a near-complete barrier from the environment, protecting internal tissues from biotic and abiotic stresses (Bargel *et al*. 2004). For instance, the cuticle can reduce pathogen (Serrano *et al*. 2014) and insect susceptibility (Eigenbrode 2002) and radiation damage (Krauss *et al*. 1997) and limit water loss (Kerstiens 1996; Riederer and Schreiber 2001). Approximately 5–10% of water lost by well-hydrated, transpiring plants during daylight hours is estimated to be attributed to evaporation occurring through the cuticle (Taiz and Zeiger 2010). However, essentially all water lost at night, and under water-limiting conditions where stomata are closed, is lost via cuticular evaporation, or through stomatal pores that are not completely sealed (Schuster *et al*. 2017; Resco de Dios *et al*. 2019).

Leaf cuticles consist of mixtures of waxes and cutin, although their structure and composition vary between and within species and developmental stages (Goodwin and Jenks 2005; Buschhaus *et al*. 2007; Pollard *et al*. 2008; Fernández *et al*. 2016). Cuticular waxes and cutin are organized into 3 layers. The deepest layer, which is continuous with the cellulosic wall, is called the cuticular layer, consisting of polysaccharides and cutin. The middle layer, or cuticle proper, is a highly hydrophobic matrix of cutin embedded with intracuticular waxes. The outermost layer is a film of epicuticular waxes (Albersheim *et al*. 2010; Domínguez *et al*. 2011). Several studies have shown that waxes are the main cuticle component that prevents nonstomatal water loss (Schönherr and Riederer 1989; Leide *et al*. 2007; Bi *et al*. 2017), serving as a barrier to water diffusion across the leaf (Seufert *et al*. 2021).

Cuticular waxes consist of solvent-extractable compounds including very long-chain fatty acids (FAs), alcohols [including primary alcohols (PAs)], hydrocarbons (HCs), aldehydes (ADs), wax esters (WEs), and alicyclics (ACs) (Jetter *et al*. 2018). In contrast, cutin is an insoluble polymer mainly formed by ester-bonded C16 and C18 hydroxy and hydroxy-epoxy FA monomers (Kolattukudy 1970). Interspecies comparisons and mutant characterization have provided evidence that leaf water conductance is determined by neither the total amount of wax nor the thickness of the cuticle but rather by the relative proportions and organization of the waxes (Riederer and Schreiber 2001). Cuticular waxes are formed in the endoplasmic reticulum (ER), where CoA thioesters of plastidial FAs are elongated to very long-chain acyl-CoAs by the multienzyme FA elongase complex (Jenks *et al*. 1995). Very long-chain acyl-CoAs are either hydrolyzed to become long-chain free FAs (Lü *et al*. 2009) or may enter 1 of 2 pathways: an acyl reduction pathway that produces PAs and WEs or an alkane-forming pathway that produces ADs, alkanes, secondary alcohols, and ketones (Yeats and Rose 2013). Waxes are delivered to the plasma membrane via vesicular transit through the Golgi (McFarlane *et al*. 2014) and across the plasma membrane by ABC transporters (McFarlane *et al*. 2010). The transport of waxes across the plasma membrane is also facilitated by glycosylphosphatidylinositol-anchored lipid transfer proteins (DeBono *et al*. 2009; Lee *et al*. 2009; Kim *et al*. 2012).

The underlying genetics of cuticular wax biogenesis and transport has been initially advanced by studying plant mutants. For example, the characterization of “glossy mutants” in grasses, including sorghum [*Sorghum bicolor* (L.) Moench], barley (*Hordeum vulgare* L.), and maize (*Zea mays* L.), has revealed some genes that are critical for wax biosynthesis and deposition (Bianchi *et al*. 1979; Jenks *et al*. 1994; Schnable *et al*. 1994; Lundqvist and Lundqvist 2008). In maize, more than 30 cuticular wax mutants have been identified, showing a glossy leaf phenotype (Schnable *et al*. 1994); however, only some of these genes have been cloned and characterized. Genes associated with glossy phenotypes, including *GLOSSY1* (*GL1*), *GL2*, *GL4*, *GL8*, and *GL26*, encode enzymes predicted to be involved in cuticular wax biosynthesis (Tacke *et al*. 1995; Xu *et al*. 2002; Dietrich *et al*. 2005; Sturaro *et al*. 2005; Fan 2007; Liu *et al*. 2009). Furthermore, *GL3* and *GL15* encode MYB and AP2-like transcription factors, respectively, regulating cuticular wax biosynthesis (Moose and Sisco 1994; Liu *et al*. 2012). Contrastingly, *GL6* and *GL13* encode the DUF538 protein and an ABC transporter, respectively, involved in wax transport (Li *et al*. 2013, 2019), while *GL14* encodes a putative membrane-associated protein with a yet-to-be-discerned function (Zheng *et al*. 2019).

While many genes involved in the biosynthesis, transport, and deposition of waxes have been reported, studies at the genome-wide level have been limited to investigating the genetic control of natural variation for the abundances of cuticular waxes in camelina [*Camelina sativa* (L.) Crantz] and sorghum (Luo, Tomasi *et al.* 2019; Elango *et al*. 2020; Jin *et al*. 2020). Our prior studies in maize have primarily focused on identifying candidate genes associated with cuticle-dependent water loss (Lin *et al*. 2020, 2022), a phenomenon we term adult leaf cuticular conductance (*g*_c_) (Lin *et al*. 2020). By integrating findings from both genome- and transcriptome-wide association studies (GWAS and TWAS) via the Fisher's combined test (FCT) in a panel of 310 maize inbred lines, Lin *et al*. (2022) revealed the association of *g*_c_ with 22 plausible candidate genes, including those predicted to play roles in cuticle precursor biosynthesis and export, intracellular membrane trafficking, and the regulation of cuticle development. With a subset of ∼50 lines having the highest and lowest *g*_c_ scores, Lin *et al*. (2022) showed that leaf cuticular wax composition of these lines is moderately predictive of their *g*_c_ values, implying that levels of specific cuticular waxes affect cuticle permeability.

To enrich the depth of our initial efforts, we combined GWAS and TWAS results to thoroughly analyze natural variation in the abundance of 60 leaf cuticular wax traits in the complete panel of 310 maize inbred lines that Lin *et al*. (2022) scored for *g*_c_. Our specific aims were to (1) determine the relationship of cuticular waxes to *g*_c_, (2) identify candidate genes associated with wax traits, (3) detect genomic hotspots that control multiple wax traits, and (4) reveal candidate genes shared between waxes and *g*_c_.

## Materials and methods

### Plant materials and experimental design

Cuticular wax data were collected from a set of 323 maize inbred lines from the Wisconsin diversity (WiDiv) panel (Hansey *et al*. 2011) planted at 2 different times (May and June 2018) in the same field at the University of California San Diego, San Diego, CA, in 2018. Leaves analyzed for wax composition were from the same plants used for *g*_c_ analysis by Lin *et al*. 2022, although they were not the same leaves (see next section for more details). The RNA-seq analysis (Lin *et al*. 2022) was performed on excised cuticle maturation zones of developing adult leaves from plants in the same plots where mature leaves were later used for wax and *g*_c_ analysis. Line selection and the design of the field experiment in both environments are further described in Lin *et al*. (2022). In both environments, the field design was augmented by including the maize inbred line Mo17 as a check plot within each incomplete block to help account for spatial variation in the field trial.

### Analysis of cuticular waxes

Due to variability in the number of leaves produced by different inbred lines and the timing of the transition from juvenile to adult leaf production, we analyzed cuticular waxes in leaves associated with the uppermost ear or the one immediately above or below it (which were always adult, nonflag leaves), instead of collecting a specific leaf number. Leaf samples for wax analysis were collected from the same plants used for *g*_c_ analysis by Lin *et al*. (2022) within a week following the *g*_c_ analysis, which was conducted during pollen shed to standardize the developmental stage. For wax analysis, a 15-cm strip lacking pathogen or other obvious damage was excised from the middle third of the primary ear leaf, or the leaf immediately above or below it, from 4 plants in each plot. Typically, the leaf sampled for wax analysis was the one immediately above or below the leaf removed earlier for *g*_c_ analysis. The samples were wrapped in wet paper towels and shipped on wet ice to Algoma University for wax analysis. Upon arrival, samples were stored at −20°C until use. For the wax analysis, leaves were thawed overnight at 4°C. Excess water was removed by gently pressing both sides of the leaf on a paper towel, and the leaves were then dissected with a scalpel to obtain an 8-cm-long segment from the middle portion, removing the midrib. The resulting 2 pieces were then photographed alongside a ruler to determine the surface area using ImageJ software (Abràmoff *et al*. 2004). Cuticular waxes were extracted by submerging the mature leaf tissue in chloroform for 60 s with gentle agitation. Three internal standards, including n-tetracosane (24:0 alkane), 1-pentadecanol (15:0 -OH), and heptadecanoic acid (17:0), were added to the extract (5 µg each). Extracted wax samples were evaporated under a gentle stream of nitrogen, derivatized to form trimethylsilyl ester and ether derivatives, resuspended in hexanes and analyzed by gas chromatography with flame ionization detection (GC-FIDs; 2 Thermo Scientific TRACE 1300 GC-FID systems were used) as described in Bourgault *et al*. (2020). Supplementary Table 1 summarizes the instrumental limits of detection and quantification for representative standards. These parameters were calculated from calibration curves using commercially available standards (Shrivastava and Gupta 2011).

Cuticular waxes with 0 values (i.e. compound not detected) in over 40% of the WiDiv panel were excluded from the analysis to mitigate the risk of biased results (Lubin *et al*. 2004). For the raw data of each retained wax trait (Supplementary File 1), abundance (µg·dm^−2^) was approximated for each 0 value by imputing a uniform random variable ranging from 0 to the lowest value quantified for a given compound within each environment (Supplementary Fig. 1; Supplementary Table 2) (Lubin *et al*. 2004; Lipka *et al*. 2013).

To screen cuticular wax traits for significant outliers, a mixed linear model was fitted for each trait in ASReml-R version 3.0 (Gilmour *et al*. 2009). The model included the grand mean and the Mo17 check as fixed effects, and genotype, environment, genotype-by-environment (G × E) interaction, incomplete block nested within the environment, column position in the field plot grid layout nested within the environment, GC-FID instrument, and GC-FID column nested within GC-FID instrument as random effects. The Studentized deleted residuals (Neter *et al*. 1996) generated from the mixed linear model were used to identify and remove outliers at a Bonferroni-corrected threshold of *α* = 0.05. For each outlier-screened wax trait, the above model was fitted to estimate the variance components for calculating heritability on a line-mean basis (Supplementary Table 3) following Lin *et al*. (2020). A best-fit model (Supplementary Table 4) was selected for each outlier-screened phenotype based on an iterative mixed model fitting procedure in ASReml-R version 3.0 from which best linear unbiased predictors (BLUPs) were generated for each wax trait (Supplementary Table 5). The BLUPs for *g*_c_ and flowering time (days to anthesis; Supplementary Table 5) were obtained from a previous study of the identical subset of the WiDiv panel in the same 2 San Diego environments (Lin *et al*. 2022). The degree of association between untransformed BLUPs of wax traits and *g*_c_ was estimated using the Pearson's correlation coefficient with the “cor.test” function in R version 3.5.1 (R Core Team 2019).

### Associations between cuticular wax traits and *g*_c_

To assess the predictive ability of *g*_c_ using 60 cuticular wax traits, random forest (RF) models were fitted using 310 inbred lines in the WiDiv panel following that of Lin *et al*. (2022). Briefly, forests were grown with the “cforest” function from the R package “party” version 1.3-7 (Strobl *et al*. 2007). Five-fold cross-validation was performed 50 times to evaluate the mean predictive ability for *g*_c_ as described in Lin *et al*. (2020). Variable importance measures were calculated using the “varimp” function in the “party” package. Parameters used for the number of trees grown (*ntree* = 1000) and predictors sampled (*mtry* = 10) were those that maximized predictive ability.

### Genomic data sets for association analyses

A set of 9,715,072 SNPs in B73 RefGen_v4 coordinates (Lin *et al*. 2022) for 310 lines with cuticular wax values were used to conduct GWAS. Briefly, a reference SNP genotype set (Wu *et al*. 2021) derived from maize HapMap 3.2.1 (Bukowski *et al*. 2018) was imputed based on a target GBS SNP set via BEAGLE v5 (Browning *et al*. 2018). Biallelic SNPs with MAF ≥ 5% and predicted dosage *r*^2^ (DR2) ≥ 0.80 were retained for GWAS (Supplementary Files 2–11).

Transcript abundances for 20,013 genes across 310 lines (Lin *et al*. 2022) were used for TWAS. The Lexogen QuantSeq 3′ mRNA-sequencing data were generated from the proximal, immature, and actively growing section of unexpanded adult leaves (sheath length < 2 cm) using an Illumina NextSeq 500 (Illumina, San Diego, CA, USA). For each gene, BLUPs were calculated to combine expression levels from the 2 environments with a mixed linear model similar to that used for wax traits. However, in this case, the physical lane on an Illumina flow cell was included as a random effect, and the GC-FID terms were not included. The probabilistic estimation of expression residuals (PEER) (Stegle *et al*. 2010) approach was applied to the matrix of BLUP expression values to account for inferred confounders. The resultant PEER values after extracting 20 latent factors were further filtered for outliers using Studentized deleted residuals and then used for conducting TWAS (Supplementary File 12).

### Genome-wide association study

To reduce heteroscedasticity and nonnormality of residuals that could lead to spurious association signals, the Box–Cox power transformation (Box and Cox 1964) was applied to each wax trait with an intercept-only model to choose the optimal value of convenient lambda (Supplementary Table 6) for transforming the BLUP values with the MASS package version 7.3-51.4 in R version 3.5.1 (R Core Team 2019). An additional outlier removal was performed using Studentized deleted residuals to generate the outlier-screened transformed BLUP values (Supplementary Fig. 2; Supplementary Table 7) of wax traits for association analyses.

With the outlier-screened transformed BLUP values of each cuticular wax trait, associations were tested with each of the 9,715,072 SNPs using a mixed linear model (Zhang *et al*. 2010) in the R package GAPIT version 3.0 (Lipka *et al*. 2012) according to Lin *et al*. (2022). The mixed linear model included days to anthesis BLUPs, principal components based on SNP genotype data, and a kinship matrix (Supplementary File 13) to control for variation in maturity, population stratification, and unequal relatedness, respectively. Principal components and the kinship matrix were calculated as described in Lin *et al*. (2022). The optimal model for GWAS selected based on the Bayesian information criterion (Schwarz 1978) included only the kinship matrix for nearly all wax traits, except for the models of 2 traits (FA 20:0 and FA 22:0) that also included days to anthesis as a covariate. A likelihood-ratio-based *R*^2^ statistic (*R*^2^_LR_) (Sun *et al*. 2010) was used to approximate the amount of phenotypic variation explained by a SNP for a transformed wax trait.

### Transcriptome-wide association study

For each of the wax traits (outlier-screened transformed BLUPs), TWAS was performed with PEER values for each of the 20,013 expressed genes to identify strong associations between trait and gene expression levels. A mixed linear model with the same kinship matrix (Supplementary File 13) and covariates from GWAS was fitted using the *gwas* function with the P3D function set to FALSE in the R package “rrBLUP” version 4.6 (Endelman 2011).

### Fisher's combined test

To integrate GWAS and TWAS results, the FCT was performed as described in Kremling *et al*. (2019). Briefly, for each wax trait, the top 10% most associated SNPs (971,508) from GWAS were assigned to the nearest gene. The *P*-values of unexpressed genes not tested in TWAS were set to 1. This allowed GWAS-identified genes to be included in the FCT analysis, thereby increasing the total number of genes tested compared to TWAS. The paired GWAS and TWAS *P*-values for each gene were used to conduct a FCT with the sumlog method in the R package “metap” (Dewey 2019). If multiple SNPs were attributed to a single gene, only the most significant SNP-gene pair was retained.

### Candidate gene identification

Because of the different statistical power and model structures in GWAS, TWAS, and FCT, potential candidate genes were selected based on the rankings of *P*-values for each statistical method as described in Kremling *et al*. (2019). To identify candidate genes from the GWAS results, the top 0.002% of SNPs were used to declare loci associated with each wax trait following that of Wu *et al*. (2021). Given that genome-wide LD decayed to nominal levels by ∼200 kb in the WiDiv panel (Lin *et al*. 2020), a ±200-kb search interval centered by the peak SNP for each declared locus was applied for candidate gene identification according to Lin *et al*. (2020). For each wax trait, the top 0.25% associated genes were selected from TWAS and FCT results based on their *P*-values. On average, GWAS, TWAS, and FCT identified comparable numbers of candidate genes for each wax trait.

### Identification of GWAS hotspots

To identify genomic regions containing multiple GWAS signals for several wax traits or “GWAS hotspots,” genome-wide scans were performed with the set of GWAS-identified loci within (5–15 traits) and across (59 traits not including total wax) 6 wax trait classes: PA (7 compounds and 1 sum trait), FA (11 compounds and 1 sum trait), HC (9 compounds and 1 sum trait), AD (4 compounds and 1 sum trait), WE (14 compounds and 1 sum trait), and AC (8 compounds and 1 sum trait). To generate a reference distribution for the number of detected GWAS loci across the genome, we implemented a sliding window procedure that used a window size of 200 kb and step size of one-fifth of the window size to approximate the number of loci (i.e. peak SNPs) within (intraclass) and across (interclass) the 6 wax trait classes. A 95% quantile of the resultant distributions indicated that an intraclass GWAS hotspot should contain at least 3 GWAS-identified loci, and an interclass GWAS hotspot should have at least 4 GWAS-identified loci. The physical boundaries of a GWAS hotspot were defined based on the entirety of all partially overlapping windows that satisfied the above criteria. A hotspot was considered to be interclass only if it included traits from 2 or more wax classes. Window sizes of 10 and 50 kb produced nearly equivalent results. The positions of GWAS hotspots in the maize genome were plotted in the R package “ChromoMap” version 4.1.1 (Anand and Rodriguez Lopez 2022).

## Results

### Phenotypic analysis

**Fig. 1.**
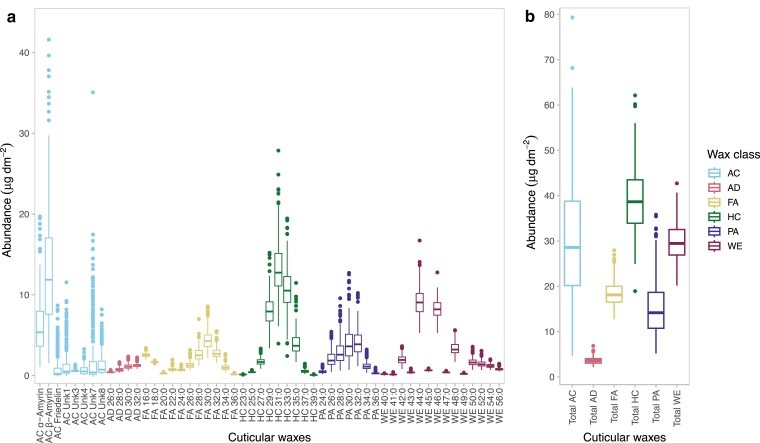
Box plots of untransformed BLUP values of cuticular waxes (µg·dm^−2^) in the maize WiDiv panel. a) Box plots showing the abundance of each wax compound. b) Box plots showing the total abundance of each wax class, including AC, AD, FA, HC, PA, and WE. Box limits indicate the upper and lower quartiles; center lines in boxes indicate the median value; whiskers indicate 1.5× interquartile range; and dots indicate outliers.

The abundance of 53 cuticular wax compounds (7 PAs; 11 FAs; 9 HCs; 4 ADs; 14 WEs; and 8 ACs) and 7 summed traits (total mass per unit area of compounds in each of the 6 classes and the total mass of all compounds per unit area) were assessed across 310 genetically diverse maize inbred lines of the WiDiv panel (Supplementary Table 3). The measured cuticular wax components showed a wide range of abundances, with the average of untransformed BLUP values ranging from 0.07 (HC 39:0) to 13.25 (HC 31:0) µg·dm^−2^ (Fig. 1). Of the 6 class sum traits, total HC was the most abundant (39.12 µg·dm^−2^), whereas total AD was the least abundant (3.63 µg·dm^−2^). However, total AC had a coefficient of variation (CV) of 0.44, which was the highest CV observed among the 6 sum traits. The 60 cuticular wax traits had an average heritability on a line-mean basis of 0.70, ranging from 0.17 (FA 18:0) to 0.97 (AC Friedelin, AC Unk1, and AC Unk7) (Supplementary Table 3), indicating that a predominant proportion of the phenotypic variation among lines can be attributed to genetic variation in this association panel. On average, the pairwise Pearson's correlations for the untransformed BLUPs of the 53 cuticular wax compounds revealed moderately strong correlations (average *r* = 0.34) between compounds belonging to the same class. In contrast, correlations were relatively weaker (average *r* = 0.12) between compounds belonging to different classes (Supplementary Fig. 3; Supplementary Table 8). Altogether, the stronger correlations observed between compounds of the same class suggest a shared genetic architecture, possibly indicating a common biosynthetic pathway.

### Prediction of *g*_c_ with cuticular waxes

Lin *et al*. (2022) assessed cuticular conductance (*g*_c_), a measure of water loss resulting from leaf cuticle permeability, in leaves from the same plants whose wax profiles are reported here (see Materials and Methods). Recognizing that the content and composition of leaf cuticular waxes may impact cuticle permeability, we investigated the relationship between *g*_c_ to the 60 cuticular wax traits. We found significant Pearson's correlations of 18 cuticular wax traits (including total wax and a subset of FAs, HCs, WEs, and ACs) with *g*_c_ (|*r*| = 0.13–0.24; *P* < 0.05). Among these 18 wax traits, only HC 27:0 showed a positive correlation with *g*_c_ (*r* = 0.14; Supplementary Fig. 3; Supplementary Table 8). Interestingly, all 10 significant WEs had a negative correlation with *g*_c_ (Supplementary Table 8), suggesting that a higher abundance of these WEs could potentially reduce the permeability of the leaf cuticle to water.

**Fig. 2.**
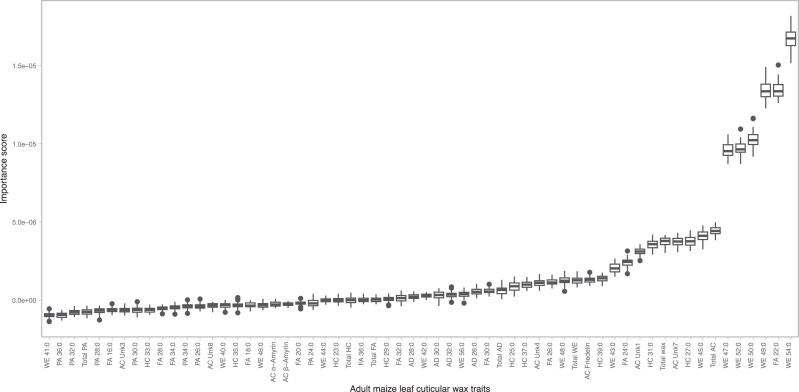
Box plots of importance scores for cuticular wax composition in RF regression to predict maize leaf cuticular conductance (*g*_c_). The wax classes are as follows: AC, AD, FA, HC, PA, and WE. Box limits indicate the upper and lower quartiles; center lines in boxes indicate the median value; whiskers indicate 1.5× interquartile range; and dots indicate outliers.

To provide a more comprehensive assessment of the relationship between *g*_c_ and leaf cuticular waxes, an RF analysis was performed to assess the collective ability of wax traits to predict *g*_c_ and determine their individual importance in the prediction model. The 60 cuticular wax traits, when combined, demonstrated an overall predictive ability of 0.27 for *g*_c_. Comprising predominantly high molecular weight WEs, only 10% of the top-ranked features (in descending order of importance: WE 54:0; FA 22:0; and WEs 49:0, 50:0, 52:0, and 47:0) contributed the majority of the predictive power, as indicated by their importance scores (Fig. 2). Suggesting stability in the RF analysis, we observed a robust positive correlation (*r* = 0.48; *P* = 1.03 × 10^−4^) between the ranks of importance scores for cuticular waxes in this analysis and a comparable analysis undertaken by Lin *et al*. (2022) using a subset of 51 WiDiv lines with extremely high or low *g*_c_ values (Supplementary Table 9). Interestingly, we observed a nonsignificant, weak negative correlation between the importance scores and the average abundance of leaf cuticular waxes (*r* = −0.17; *P* = 0.22). This suggests that the individual wax traits with the highest average abundances across the WiDiv panel are not necessarily the most important for predicting *g*_c_. While the 60 wax traits collectively offered only moderate predictive ability (0.27) for *g*_c_, a genetic dissection of wax composition would contribute to a deeper understanding of their genetic regulation and their role in influencing *g*_c_ variability.

### Quantitative genetic analysis for identification of candidate genes for cuticular waxes

We performed GWAS and TWAS on the transformed BLUPs of the 60 cuticular wax traits to investigate the genetic basis of cuticular wax accumulation in the adult maize leaf and then integrated the GWAS and TWAS results with the FCT to enhance statistical power. A total of 6,186 unique candidate genes (top 0.002% SNPs) were identified from GWAS (Supplementary Tables 10 and 11), 1,956 (top 0.25% genes) from TWAS (Supplementary Table 12), and 2,965 (top 0.25% genes) from FCT (Supplementary Table 13) across the 60 wax traits. The number of unique candidate genes identified across the 3 methods varied from 103 (AC α-Amyrin and AC β-Amyrin) to 398 (AD 30:0) for each of the 60 traits (Supplementary Table 14). Of the identified candidate genes, 231 were designated as “higher confidence” candidates (Supplementary Table 15) because they were detected by FCT, TWAS, and GWAS when collectively considering all 60 wax traits. Of these, 69 (∼30%) were associated with 1 or more of the 6 wax compounds that were ranked by the RF analysis as being of the highest importance in predicting *g*_c_ (in descending order of importance: WE 54:0; FA 22:0; and WEs 49:0, 50:0, 52:0, and 47:0). As shown in Supplementary Table 15, the higher confidence candidate genes were divided into 3 groups using the following criteria: group 1 consisted of 41 genes identified by FCT, TWAS, and GWAS for the same wax compound(s); Group 2 comprised 42 genes not meeting criteria of group 1 but associated via FCT, TWAS, and GWAS with members of the same wax class(es); and group 3 included 148 genes not meeting the criteria for groups 1 and 2 but identified by all 3 methods (i.e. associated with waxes belonging to different classes by different methods). Of the 231 higher confidence candidates listed in Supplementary Table 15, 11 genes are featured as plausible candidates in Table 1 based on predicted functions that can be related to cuticular waxes. Five of these (*Zm00001d045660*, *Zm00001d018474*, *Zm00001d038404/Zm00001d038405*, *Zm00001d013798*, and *Zm00001d049100*) were associated with at least 1 of the 6 compounds found to be most important for the *g*_c_ trait via RF analysis. The closest *Arabidopsis* [*Arabidopsis thaliana* (L.) Heynh.] and rice (*Oryza sativa* L.) homologs of these 11 genes along with additional details are listed in Supplementary Table 16.

**Table 1.** Plausible candidate genes identified for adult maize leaf cuticular waxes via a GWAS, TWAS, FCT, and GWAS hotspot analysis in the maize WiDiv panel.

Gene ID	Identity/predicted function	Chr.	Group*^a^*	GWAS hotspot class*^b^*
Zm00001d049100	Target of Myb1-like (TOL) protein	4	Group 1: PA	Intra: PA and WE; Inter: PA, WE, and AD
Zm00001d013301	Sec23/Sec24 transport family protein (SAG4)	5	Group 3	
Zm00001d013798	Cycloartenol synthase (CAS)	5	Group 1: AC	Intra: AC
Zm00001d017941	3-Ketoacyl-CoA synthase (KCS)	5	Group 1: WE	
Zm00001d018474	GDSL esterase/lipase (GELP2)	5	Group 3	
Zm00001d038404/Zm00001d038405*^c^*	Ypt/Rab-GAP	6	Group 3	
Zm00001d019104	Exocyst (EXO) complex component	7	Group 3	
Zm00001d045660	3-Ketoacyl-CoA synthase (KCS)	9	Group 1: PA, FA, and HC	
Zm00001d046642	GDSL esterase/lipase (OSP1 homolog)	9	Group 2: FA	
Zm00001d024723	Long chain acyl-CoA synthetase (LACS; CER8)	10	Group 3	
Zm00001d026600	Protein S-acyltransferase (PAT38)	10	Group 1: PA	Intra: PA

An expanded version of this table with additional information about each gene is provided as Supplementary Table 16.

^*a*^Group 1, genes identified by FCT, TWAS, and GWAS for the same trait; group 2, genes not meeting criteria of group 1 but associated via FCT, TWAS, and GWAS with members of the same wax class(es); group 3, genes not meeting the criteria for groups 1 and 2 but associated with 1 or more wax traits by all 3 methods. The wax classes are as follows: AC, AD, FA, HC, PA, and WE.

^*b*^GWAS hotspots for traits within (intra) and across (inter) wax classes.

^*c*^In B73 RefGen_v4, this gene was erroneously split into the 2 gene models as indicated, but in B73 RefGen_v5, they have been correctly merged into a single gene model with identifier Zm00001eb289920.

**Fig. 3.**
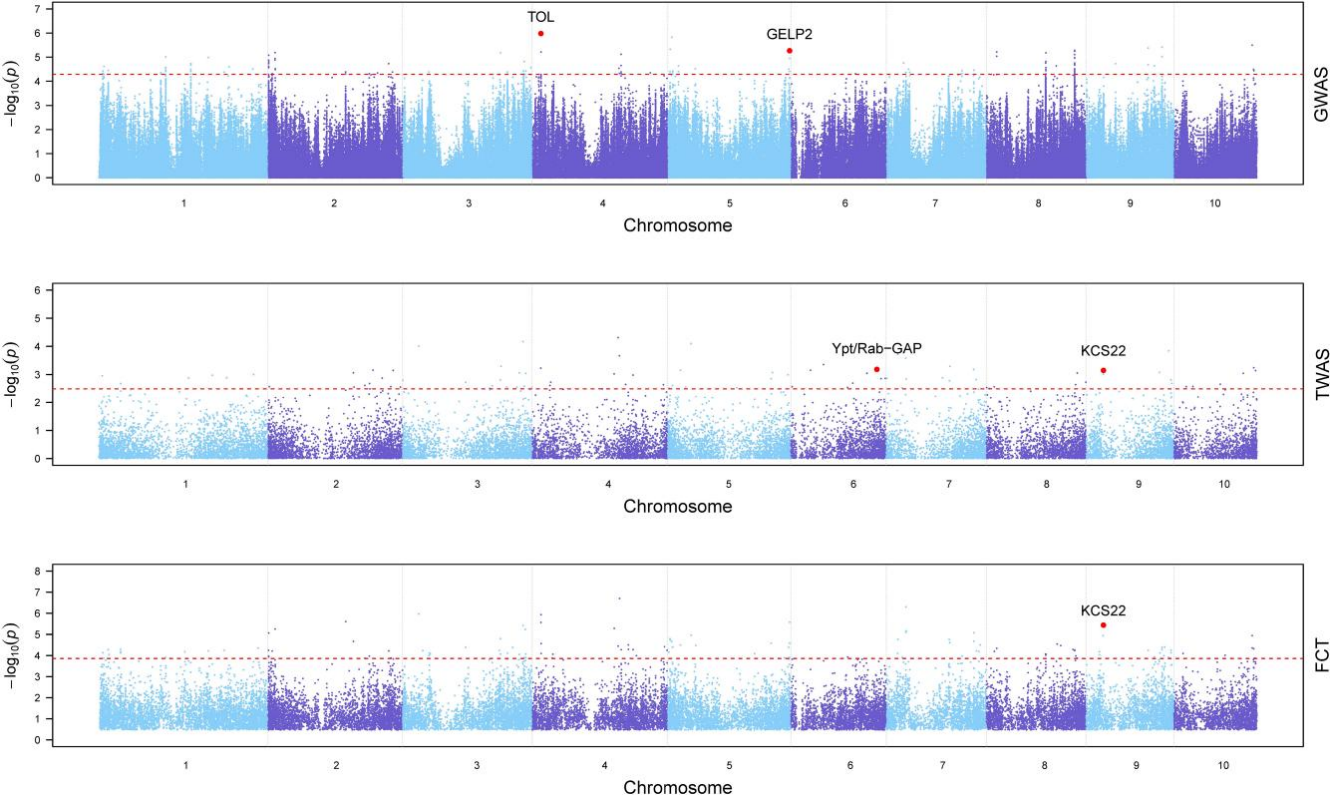
Manhattan plots of GWAS, TWAS, and FCT results for WE 49:0. Each point represents a SNP or gene with its −log_10_  *P*-value (*y*-axis) from GWAS, TWAS, and FCT plotted as a function of physical position (Mb, B73 RefGen_v4) across the 10 chromosomes of maize (*x*-axis). Horizontal dashed lines indicate the thresholds of top 0.002%, top 0.25%, and top 0.25% for GWAS, TWAS, and FCT, respectively. Plausible candidate genes (Table 1) that are within 200 kb of a top 0.002% GWAS peak SNP or ranked in the top 0.25% in TWAS or FCT are highlighted with larger dots and labeled in the Manhattan plots.

Six of the plausible candidate genes listed in Table 1 are predicted or functionally confirmed in maize to have a role in cuticular wax biosynthesis (Supplementary Table 16). Two members of the 3-ketoacyl-CoA synthase (KCS) family, namely KCS22 encoded by *Zm00001d045660* (*KCS22*) and a KCS6 paralog encoded by *Zm00001d017941* (*GLOSSY4B*, *GL4B*), were identified in association with 26 and 6 unique wax traits, respectively. In TWAS and FCT, *KCS22* exhibited associations with 4 (WEs 49:0, 50:0, 52:0, and 54:0; Fig. 3) of the 6 compounds identified as most important for *g*_c_ through RF analysis. However, no significant associations with these 6 compounds were observed for *GL4B*. *Zm00001d013798*, which encodes a homolog of *Arabidopsis* CYCLOARTENOL SYNTHASE1 (CAS1) (Corey *et al*. 1993; Babiychuk *et al*. 2008), was identified in GWAS for 5 ACs and 11 unique traits (including 2 FAs and 9 ACs) when combining TWAS and FCT results. Two additional plausible candidate genes listed in Table 1, encoding putative Gly-Asp-Ser-Leu (GDSL) lipases, are each associated with 3 unique wax traits. One of these, *Zm00001d046642* (encodes a homolog of *Arabidopsis* OCCLUSION OF STOMATAL PORE1, OSP1), showed associations with FA 24:0 in TWAS and with FA 36:0 and HC 35:0 in both GWAS and FCT. The other gene, *Zm00001d018474* (*GDSL ESTERASE/LIPASE2*, *GELP2*), exhibited associations with HC 27:0 in TWAS and FCT, as well as with WEs 49:0 and 56:0 in GWAS (Fig. 3). Finally, the *CER8* gene (*Zm00001d024723*), encoding a homolog of *Arabidopsis* CER8/LONG-CHAIN ACYL-COA SYNTHETASE1 (LACS1) (Lü *et al*. 2009), was associated with PA 32:0 in GWAS and with HC 37:0 in both TWAS and FCT.

The transport of waxes from their synthesis site in intracellular membranes to the cell wall surface depends on Golgi-mediated vesicular trafficking (McFarlane *et al*. 2014). Accordingly, the remaining 5 of the 11 plausible candidate genes listed in Table 1 were chosen because they encode proteins predicted to function in intracellular trafficking. *Zm00001d038404*/*Zm00001d038405*, which encodes a protein with a putative Ypt/Rab-GTPase-activating protein (GAP) domain, was found to be associated with 9 unique traits. Notably, among these traits, 4 (WEs 49:0, 50:0, 52:0, and 54:0; Fig. 3) were found to be of high importance in the RF analysis. *Zm00001d019104*, encoding a homolog of *Arabidopsis* exocyst complex component 84B (EXO84B), was associated with FA 32:0 and HC 39:0. *Zm00001d013301* (*SALT-TOLERANCE-ASSOCIATED-GENE4*, *SAG4*) (Luo, Wang *et al*. 2019), which associated with FA 36:0 and 3 HC (33:0, 35:0, and total HC) traits, encodes a SECRETION23/SECRETION24 (SEC23/SEC24) family protein. *Zm00001d026600* (*PROTEIN S-ACYLTRANSFERASE38*, *PAT38*), which encodes a protein with similarity to members of the DHHC-type zinc finger protein family in *Arabidopsis* that mediate the S-acylation of proteins (Yuan *et al*. 2013), was found to be associated with 8 traits (6 PAs, AD 28:0, and WE 48:0). *Zm00001d049100*, encoding a protein similar to members of the *Arabidopsis* TARGET OF MYB1-LIKE (TOL) family of proteins involved in the sorting of ubiquitinated protein cargoes into multivesicular bodies (MVBs) (Winter and Hauser 2006), was associated with 11 AD, PA, and WE traits (Fig. 3).

Identification of additional candidate genes was facilitated by cross-referencing our GWAS, TWAS, and FCT results against a list of 115 candidate genes for maize leaf cuticle biosynthesis and its regulation, as compiled by Qiao *et al*. (2020) based on transcriptome analysis of cuticle maturation. This resulted in the identification of 39 candidate genes from the list of Qiao *et al*. (2020) that were associated with at least 1 wax trait (Supplementary Table 17). Four of these genes (*Zm00001d018474*, *GELP2*; *Zm00001d024723*, *CER8*; *Zm00001d045660*, *KCS22*; and *Zm00001d046642*, *OSP1* homolog) are included in our list of plausible candidate genes (Table 1), as they were detected by GWAS, TWAS, and FCT. The remaining 35 candidate genes, identified in the present study by 2 or fewer of the 3 association methods, were collectively found to be associated with a total of 32 traits from all 6 wax classes. Although these 35 candidate genes have less robust support compared to those detected by all 3 methods, they are still well-supported as candidates having a purported role in the genetic control of wax traits. In the context of the 6 top-ranked wax compounds revealed by the RF analysis as having high importance for predicting *g*_c_, 5 of the remaining 35 candidate genes (*GLYCEROL-3-PHOSPHATE ACYLTRANSFERASE20*, *GPAT20*; *KCS24*; *MYB162*; *PHOSPHOLIPID TRANSFER PROTEIN HOMOLOG1*, *PLT1*; and *PLT2*) were associated with WEs 47:0, 49:0, and/or 54:0.

### Hotspot analysis for waxes

**Fig. 4.**
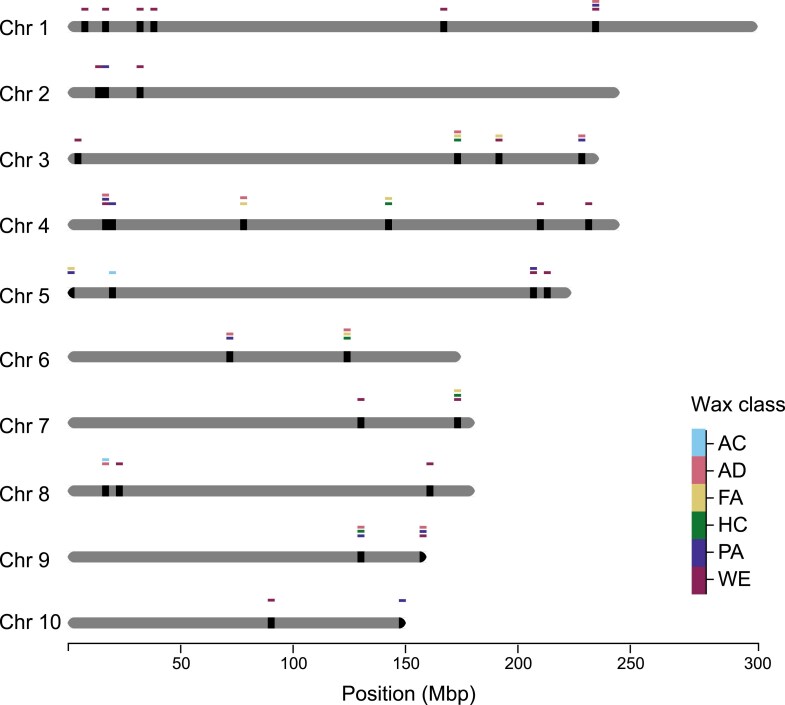
Genomic positions of GWAS hotspots in the maize genome. A chromosomal representation of GWAS hotspots was generated by plotting their physical positions (Mb, B73 RefGen_v4) across the 10 chromosomes of maize (*x*-axis). On each chromosome, rectangles indicate the positions of GWAS hotspots. Hotspots are depicted at the wax class level as follows: AC, AD, FA, HC, PA, and WE. Hotspots with stacked bars represent interclass hotspots, while those with a single bar represent intraclass hotspots.

Given that biosynthetic pathways and regulatory networks can be shared by wax compounds (Post-Beittenmiller 1996; Lewandowska *et al*. 2020), we sought to identify genomic hotspots that possessed GWAS signals for several wax traits, potentially housing genes involved in the deposition of multiple cuticular waxes. Through the implementation of a sliding window method, we identified 34 and 15 GWAS hotspots for traits within (intraclass) and across (interclass) wax classes, respectively (Fig. 4; Supplementary Table 18). With the exception of FA, each of the wax classes had at least 1 intraclass hotspot. The PA and WE classes had the highest number with 10 and 20 intraclass hotspots, respectively. Notably, 65% of the WE hotspots were associated with 1 or more of the 5 WEs (47:0, 49:0, 50:0, 52:0, and 54:0) found to be most important for predicting *g*_c_ in the RF analysis. Of the 15 interclass hotspots, 9 were coincident (i.e. at least 50% overlapping intervals) with 12 of the intraclass hotspots (Fig. 4; Supplementary Table 18). Among the 15 interclass hotspots identified, 6 were associated with 1–5 WEs, along with 1–6 additional traits drawn from up to 2 other wax classes (AD, PA, HC, and/or FA).

The combined set of intra- and interclass GWAS hotspots contained 109 unique loci associated with 45 traits that spanned all 6 wax classes. Collectively, these hotspots contain 371 unique genes. Of these, 33 were also identified by TWAS, and 110 by FCT, but not necessarily for the same traits associated via GWAS (Supplementary Table 19). Twenty-one of the hotspot genes are among the 231 higher confidence candidates identified by all 3 methods (Supplementary Tables 15 and 19), of which 3 are listed as plausible candidates in Table 1. The 3 plausible and higher confidence candidate genes located in GWAS hotspots are as follows: *Zm00001d013798* (CAS1 homolog) in an intraclass hotspot for ACs; *PAT38* in an intraclass hotspot for PAs; and *Zm00001d049100* (TOL homolog) in intraclass hotspots for PAs and WEs (Supplementary Tables 1, 16, 18, and 19). Notably, the TOL homolog *Zm00001d049100* was located in the most trait-dense interclass hotspot we identified, which was associated with 4 PA, 1 AD, and 5 WE traits, with 4 of these WEs (47:0, 49:0, 50:0, and 52:0) identified through RF analysis as strongly predictive of *g*_c_.

We identified an additional set of 4 plausible candidate genes located within hotspots according to the relevance of their predicted functions to the abundance of cuticular waxes (Supplementary Table 19). Although these 4 genes were not found by TWAS, they were found associated with 1 or more wax traits by both GWAS and FCT. A second gene (*Zm00001d008671*) encoding a predicted CAS1 homolog was found within an intraclass hotspot for ACs and an interclass hotspot for AC and AD traits on chromosome 8 (Supplementary Table 19). Interestingly, within an intraclass hotspot for PAs on chromosome 1, 2 genes (*Zm00001d032719* and *Zm00001d032721*), ∼106 kb apart, encode homologs of FATTY ALCOHOL OXIDASE4b (FAO4b) found in *Arabidopsis* (Supplementary Table 19). Lastly, *Zm00001d049155*, a second associated SEC23/SEC24 homolog, was located in an intraclass hotspot for PAs.

### Investigating a shared genetic basis between waxes and *g*_c_

Given that the RF analysis showed a moderate predictive ability of *g*_c_ with all 60 waxes, we integrated the GWAS (0.002% SNPs), TWAS (top 0.25% genes), and FCT (top 0.25% genes) results from both this study and Lin *et al*. (2022) to explore the extent to which natural variation in leaf cuticular waxes and *g*_c_ is connected through a shared underlying genetic architecture. A total of 17 unique candidate genes were identified in GWAS, 9 in TWAS, and 10 in FCT for both *g*_c_ and at least 1 wax trait (Supplementary Table 20). Collectively, this set of 35 nonredundant genes was associated with 28 unique wax traits spanning all 6 wax classes. Among the 35 genes, only *Zm00001d017380*, encoding a homolog of *Arabidopsis* MEMBRANE STEROID BINDING PROTEIN1, was detected by more than 1 method (TWAS and FCT). The set of 35 genes included 4 identified by Lin *et al*. (2022) as plausible candidates associated with *g*_c_: *Zm00001d038404*/*Zm00001d038405* (encodes a Ypt/Rab-GAP homolog) for PA 36:0; *Zm00001d012175* (encodes PECTIN ACETYLESTERASE 5, PAE5) for HC 27:0; *Zm00001d005087* (encodes G2-LIKE-TRANSCRIPTION FACTOR 35, GLK35) for AD 30:0, total AD, AC β-Amyrin, and total wax; and *Zm00001d050185* (encodes MYB108) for HC 35:0. Collectively, the predicted functions of proteins encoded by these genes implicate the importance of cell wall biosynthesis (PAE5), transcriptional regulators of cuticle development (GLK35 and MYB108), and intracellular membrane trafficking (Ypt/Rab-GAP homolog) in the genetic control of both *g*_c_ and waxes.

## Discussion

Waxes are critical for cuticle function in protecting plants from biotic and abiotic stresses, including drought stress. Most prior studies identifying genetic determinants of cuticle biogenesis in maize and other grasses have focused on juvenile (seedling) leaves. However, the life cycle of maize is dominated by adult leaves, whose cuticular wax profiles are very different from juvenile leaves (Bourgault *et al*. 2020). In this study, we leveraged natural variation in the abundance of 60 wax traits scored on adult maize leaves belonging to 310 diverse inbred lines of the WiDiv panel. Our overarching goal was to search for genetic determinants of adult cuticular wax accumulation and to analyze the relationship between wax composition and cuticular conductance (*g*_c_) to determine which waxes are most important for protecting adult leaves from water loss across the cuticle. This study extends our prior work employing the same genetic material (adult maize leaves from the same subset of WiDiv lines) and experimental strategy (joint analysis of associations via GWAS, TWAS, and FCT) to identify genetic determinants of *g*_c_ (Lin *et al*. 2022). It also builds on our earlier study that investigated genetic determinants of *g*_c_ via GWAS alone with a larger collection of WiDiv lines (Lin *et al*. 2020).

The prevailing consensus is that the water barrier property of the cuticle is conferred mainly by waxes (Schönherr 1976; Kerstiens 2006; Isaacson *et al*. 2009; Jetter and Riederer 2016) and that wax composition, rather than quantity, is critical to this function (Vogg *et al*. 2004; Buschhaus and Jetter 2012; Jetter and Riederer 2016; Jetter *et al*. 2018). Consistent with that view, our RF analysis identified 6 wax compounds, rather than total wax, as most critical for predicting *g*_c_: 5 high molecular weight WEs (47:0, 49:0, 50:0, 52:0, and 54:0) and FA 22:0. This finding extends our earlier RF analysis employing a subset (51) of the inbred lines used here, which identified the same 6 wax compounds among 12 whose abundance best predicted *g*_c_ (Lin *et al*. 2022). The earlier analysis also identified additional compounds that were not corroborated by the larger-scale analysis presented here. We believe the present analysis is more definitive because its larger scale allowed us to use BLUPs of wax abundance—rather than the average raw abundance values used by Lin *et al*. (2022)—that were corrected for nongenetic sources of variation attributed to analytical lab and spatial field effects. Our findings from both studies, which highlight the key role for WEs in predicting *g*_c_, are consistent with research implicating WEs with carbon chains > 48:0 in the establishment of water barrier properties of the cuticle during adult maize leaf development (Bourgault *et al*. 2020). The longer the carbon chain, the higher their melting point and, consequently, their hydrophobicity (Patel *et al*. 2001), potentially explaining the importance of these WEs in waterproofing the cuticle. Further consistent with our findings, increased water permeability has been shown for *Arabidopsis wax ester synthase1* mutants with reduced WE load (Patwari *et al*. 2019). Yet, our finding that *g*_c_ variation is not strongly predicted by any feature of wax composition demonstrates that other factors are also important for the water barrier function of the cuticle.

To elucidate the genetic architecture of cuticular wax accumulation, the abundances of 60 wax traits were analyzed in this study as quantitative traits in GWAS employing ∼10 million SNPs, in TWAS investigating associations with transcript abundances of ∼20,000 genes expressed in cuticle maturation zones, and in FCT combining results of GWAS and TWAS. Intersectional approaches were employed to identify genes whose potential roles in cuticular wax accumulation are supported by multiple lines of evidence. To enhance this effort, we also identified genomic hotspots as loci associated via GWAS with more than 1 wax trait and compared lists of candidate genes identified in this study with those identified in our earlier studies searching for genetic determinants of *g*_c_ and cuticle development.

Among the 231 higher confidence candidate genes identified by GWAS, TWAS, and FCT listed in Supplementary Table 15, 6 are featured in Table 1 as plausible candidates encoding enzymes with predicted functions in cuticular wax biosynthesis. One of these, *CER8* (*Zm00001d024723*), encodes a LACS with a previously demonstrated role in the accumulation of epicuticular waxes in juvenile leaves, as shown through mutant analysis (Zheng *et al*. 2019). This gene was also identified as a candidate determinant of cuticular wax accumulation in a recent multiomic study analyzing associations between variation in gene expression and the abundance of cuticular waxes across multiple seedling organs and genotypes (Chen *et al*. 2024). LACS enzymes convert C16 and C18 FAs into activated CoA thioesters, which are precursors of all aliphatic waxes (Yeats and Rose 2013). Our study detected an association of maize *CER8* with members of both the acyl reduction (PA 32:0) and alkane-forming pathways (HC 37:0), suggesting possible involvement in both pathways, consistent with the known role of LACS enzymes.

KCS enzymes are components of FAE complexes that function downstream of LACS enzymes to elongate C16 and C18 CoA thioesters, forming VLCFAs and their derivatives, which comprise the majority of cuticular wax (Lee and Suh 2013). We identified 2 KCS enzyme genes as higher confidence candidates associated with various wax traits by all 3 methods (Table 1): *KCS22* (*Zm00001d045660*) and *GL4B/KCS5* (*Zm00001d017941*). While neither gene has an experimentally validated function in wax biosynthesis, *GL4A*, a paralog of *GL4B*, has a demonstrated role in the accumulation of juvenile epicuticular waxes (Liu *et al*. 2009). Remarkably, *KCS22* was associated with more wax traits than any other gene in our study (26 in total) and is one of the most strongly associated genes for many of these traits (Supplementary Table 15). The associations we observed for both *KCS* genes with waxes that are products of the acyl reduction and alkane-forming pathways are consistent with the established functions of KCS enzymes.

Two genes encoding GDSL lipases were identified as plausible candidates (Table 1). *GELP2* (*Zm00001d018474*), which is expressed in stomata (Sun *et al*. 2022), encodes a homolog of *Arabidopsis CUS2*, a putative cutin synthase that promotes the accumulation of cutin polymers and cuticular ridges in sepals (Hong *et al*. 2017). *GELP2* may, therefore, impact wax accumulation indirectly via a function in cutin matrix assembly. The other GDSL lipase gene we identified, *Zm00001d046642*, is a homolog of *Arabidopsis OSP1*, which is required for stomatal cuticular ledge formation but does not affect cutin accumulation (Tang *et al*. 2020). Biochemically, OSP1 functions as a thioesterase, catalyzing the formation of VLCFAs and promoting the accumulation of all classes of aliphatic cuticular waxes (Tang *et al*. 2020). Thus, the maize *OSP1* homolog gene we identified could impact wax biosynthesis directly as well.

Another class of wax biosynthetic enzyme genes identified in our study is represented by a closely spaced pair of *FAO4b* genes (*Zm00001d032719* and *Zm00001d032721*). These genes were not associated with wax traits by all 3 methods and are therefore not included in Table 1 as plausible candidate genes, but both are located in an intraclass GWAS hotspot for PAs (Supplementary Table 19). Both genes were also associated with PAs via FCT, making them well-supported candidates for PA biosynthesis. Recent work in *Arabidopsis* demonstrated that 2 FAO enzymes function in the oxidation of PAs, converting them to ADs (Yang *et al*. 2022). Thus, our finding of an association between the 2 *FAO4b* genes and variation in PA accumulation is consistent with the known functions of FAOs in *Arabidopsis*.

AC waxes (triterpenes and steroids) are synthesized independently from VLCFA-derived aliphatic waxes via the mevalonate pathway, which involves oxidosqualene cyclases (OSCs) that convert 2,3-oxidosqualene into steroids and triterpenes (Thimmappa *et al*. 2014). A putative *OSC* gene, *Zm00001d013798*, encoding a homolog of the *Arabidopsis OSC* gene *CAS1*, was identified as a plausible candidate. *Zm00001d013798* was associated with multiple AC waxes through all 3 methods and is the first or second most strongly associated gene for the majority of these wax traits via both TWAS and FCT. This gene is also located in an intraclass GWAS hotspot on chromosome 5 for multiple AC waxes. Another *Arabidopsis* CAS1 homolog, *Zm00001d008671*, was identified in a different GWAS hotspot on chromosome 8 for AC waxes (total AC, α-amyrin, and β-amyrin). While not associated with ACs or other wax traits via TWAS, *Zm00001d008671* was strongly associated with total ACs, α-amyrin, and β-amyrin via FCT. Both *OSC* genes we identified are therefore strongly supported as key genes for AC wax biosynthesis.

Based on the established role of membrane trafficking in the transport of waxes to the cell surface (McFarlane *et al*. 2014), we highlight various potential intracellular trafficking proteins as plausible candidate genes for maize cuticular wax biogenesis in Table 1. SEC23/SEC24 proteins initiate COPII-mediated vesicle transport between the ER and the Golgi apparatus (Brandizzi and Barlowe 2013; Cui *et al*. 2022). *Zm00001d013301* encodes a SEC23/SEC24 homolog named as *SAG4* based on a demonstrated role in conferring salt tolerance in maize (Luo, Wang *et al*. 2019), but it has not previously been linked to cuticles. Another SEC23/24 gene, *Zm00001d049155*, is located in a GWAS hotspot for PAs and was also associated with PA via FCT. The associations we observed between SEC23/24 homologs and cuticular waxes are particularly notable given a recent finding that a mutation in a *SEC23* gene in cucumber causes a glossy phenotype and drastically reduces cuticular wax load in fruits (Gao *et al*. 2023).

The exocyst complex functions at the plasma membrane as a vesicle tether, facilitating vesicle fusion at appropriate sites on the cell surface (Cui *et al*. 2022). A putative exocyst subunit gene, *EXO84B* (*Zm00001d019104*), was associated with 2 wax compounds from different classes, suggesting a possible role for the exocyst complex in the vesicle-mediated delivery of waxes to the outer cell surface. Rab GTPases on vesicle surfaces direct vesicle trafficking through interactions with tethering proteins on target membranes; GAPs modulate their activity (Cui *et al*. 2022). One of our plausible candidate genes, *Zm00001d038404/Zm00001d038405*, encodes a Ypt/Rab-GAP domain family protein, a putative regulator of Rab activity. Notably, another member of this gene family (*Zm00001d025180*) was identified as a candidate determinant of seedling cuticle composition by the multiomic association study of Chen *et al*. (2024) and as a candidate determinant of *g*_c_ in adult leaves by Lin *et al*. (2022). In the present study, *Zm00001d038404/Zm00001d038405* was associated with several high molecular weight WEs and was the most strongly associated gene by TWAS for 2 of them. This gene is particularly interesting because we also identified it as a plausible candidate determinant of *g*_c_ by GWAS (Lin *et al*. 2022) and because 4 of the WEs associated with this gene are among the top 6 compounds most predictive of *g*_c_. Taken together, our findings suggest that this putative Ypt/Rab-GAP impacts *g*_c_ by playing a role in the transport of high molecular weight WEs.

S-Acylation is a posttranslational modification mediating protein association with membranes (Batistič *et al*. 2008), potentially playing a role in the localization of many proteins participating in cuticle delivery to and transport across the plasma membrane. We identified an S-acyltransferase gene, *Zm00001d026600* (*PAT38*; Yuan *et al*. 2013), located in an intraclass GWAS hotspot for PAs and among the genes most highly associated with PAs by TWAS and FCT. Thus, *PAT38* is strongly supported as a key determinant of PA accumulation through an unknown mechanism involving protein S-acylation.

Another plausible candidate gene with strong, intersectional support, *Zm00001d049100*, encodes a TOL family protein with a potential role in the intracellular trafficking of cuticle lipids. *Zm00001d049100* is located in a trait-dense interclass GWAS hotspot for multiple waxes and was strongly associated with the same waxes by TWAS and FCT. TOL family proteins in *Arabidopsis* are known for their ubiquitin-binding role, which initiates the signal for cargo protein entry into the MVB pathway (Winter and Hauser 2006). Although TOL proteins have not been previously linked to cuticle formation, the MVB pathway has been implicated in cuticle formation in both *Arabidopsis* and maize (Goodman *et al*. 2021; Lin *et al*. 2022). Our findings suggest that *Zm00001d049100* may play a role in membrane trafficking processes important for the accumulation of multiple aliphatic wax classes.

A final candidate gene of particular interest, *GLK35* (*Zm00001d005087*), encodes a MYB domain-containing putative transcription factor. Although it was identified in this study only by TWAS, it is noteworthy because it was previously identified as a plausible candidate determinant of *g*_c_ via both TWAS and FCT (Lin *et al*. 2022). Remarkably, *GLK35* was the gene most strongly associated with *g*_c_ via TWAS, underscoring its probable influence on *g*_c_, albeit through effects on other waxes besides WEs, as indicated by its associations with AC β-Amyrin, AD 30:0, total AD, and total wax via TWAS in the present study. Notably, the 2 closest relatives of *GLK35* in *Arabidopsis* are genes encoding the recently characterized MYB-SHAQKYF transcription factors, MYS1 and MYS2 (Lin *et al*. 2022; Liu *et al*. 2022). These redundant transcriptional repressors directly suppress the expression of *DECREASE WAX BIOSYNTHESIS*, another transcriptional regulator of wax biosynthesis. Analysis of *mys1mys2* double mutants shows that these genes promote wax biosynthesis (especially of HCs, the most abundant wax class in *Arabidopsis* leaves), protect leaves from water loss in a detached leaf assay, and confer drought tolerance (Liu *et al*. 2022). The relationship to *MYS1* and *MYS2* strengthens the hypothesis that *GLK35* acts as a determinant of *g*_c_ via regulation of wax biosynthesis genes.

Only 2 of the 11 genes identified in Table 1 as plausible candidate determinants of cuticular wax abundance are among the 21 plausible candidate *g*_c_ determinants identified by Lin *et al*. (2022): the Ypt/Rab-GAP domain protein encoded by *Zm00001d038404/Zm00001d038405* and the putative transcription factor *GLK35* encoded by Z*m00001d005087*, discussed earlier. Since the only difference between these 2 studies is the traits being analyzed, this lack of overlap likely reflects the role of other factors besides wax composition in *g*_c_, consistent with our finding from RF analysis that all cuticular wax traits combined predict only 0.27 of the variation in *g*_c_. Moreover, with the exception of the *CER8* gene (*Zm00001d024723*), encoding a putative LACS enzyme discussed earlier, none of the 231 higher confidence candidate genes listed in Supplementary Table 15 have been previously demonstrated by genetic evidence to play a role in cuticle formation. Thus, none of our higher confidence candidates correspond to *GLOSSY* genes with known identities. However, the *GL26* gene (*Zm00001d008622*), encoding a FAE complex component (ECR/enoyl-CoA reductase; Fan 2007), was associated with WE 43:0 via GWAS (Supplementary Table 11) and with AD 28:0 via FCT (rank 45; Supplementary Table 13). It is also among the 39 candidates listed in Supplementary Table 17 based on the overlap between genes associated with at least 1 trait in our study and top candidates for regulation of cuticle development identified by Qiao *et al*. (2020) via transcriptome analysis. The 2 *KCS* genes we identified as plausible candidates (*KCS5*/*GL4B* and *KCS22*) differ from the 3 *KCSs* with genetic evidence for a function in cuticular wax accumulation (*KCS23/GL4*, Liu *et al*. 2009; *KCS19/AD1*, Liu *et al*. 2021; and *KCS12*, Xu *et al*. 2024). Our plausible candidate *KCS* genes 5 and *22* also differ from a *KCS* gene (*KCS24*) associated with cuticle-dependent seedling traits via GWAS by Xu *et al*. (2024) and with cuticular wax variation across multiple seedling organs by Chen *et al*. (2024). This lack of correspondence most likely reflects our focus on adult leaves, whereas the other studies focused on juvenile (seedling) leaves, which have distinct cuticular wax profiles (Bourgault *et al*. 2020). Given the importance of adult leaves for agronomically significant traits, we believe our study has identified many new candidate genes that could ultimately be valuable for breeding or engineering maize plants with improvements in cuticle-dependent traits of agronomic value.

## Supplementary Material

jkae241_Supplementary_Data

## Data Availability

All raw 3′-mRNA-seq data are available from the NCBI Sequence Read Archive under BioProject PRJNA773975. Supplementary Files 1–13 are available on CyVerse DOI 10.25739/4x7e-d439 (Lin 2024) (https://datacommons.cyverse.org/browse/iplant/home/shared/commons_repo/curated/Lin_CuticularWaxGWASTWAS_2024). Code is available from GitHub (https://github.com/GoreLab/Maize_Cuticular_Wax). Supplemental material available at G3 online.
