# Supplementary material for: Integrative multiomic analysis identifies genes associated with cuticular wax biogenesis in adult maize leaves
Source: G3 (Bethesda). 2024 Oct 10;14(12):jkae241. doi: 10.1093/g3journal/jkae241 (PMC11631437; doi:10.1093/g3journal/jkae241)
Supplement: jkae241_Supplementary_Data [file jkae241_supplementary_data.zip › Supplemental_Material_Legends_G3-2024-405336.docx]

### **Supplemental Material**

**Figure S1.** Histogram plots of raw data values, both before outlier removal and after the addition of random uniform variables, for cuticular waxes in the maize Wisconsin diversity panel. The x-axis shows wax abundance (μg dm⁻²), while the y-axis depicts the frequency of each wax abundance. Each histogram plot corresponds to a specific wax trait. The different wax classes are as follows: alicyclic (AC), aldehyde (AD), fatty acid (FA), hydrocarbon (HC), primary alcohol (PA), and wax ester (WE).

**Figure S2.** Box plots of transformed best linear unbiased predictor (BLUP) values of cuticular waxes in the maize Wisconsin diversity panel. (a) Box plots showing the abundance of each wax compound. (b) Box plots showing the total abundance of each wax class, including alicyclic (AC), aldehyde (AD), fatty acid (FA), hydrocarbon (HC), primary alcohol (PA), and wax ester (WE). Box limits indicate the upper and lower quartiles; center lines in boxes indicate the median value; whiskers indicate 1.5x interquartile range; and dots indicate outliers.

**Figure S3.** Correlation matrix for untransformed best linear unbiased predictors (BLUPs) of adult maize leaf cuticular conductance (*g*_c_; g∙h^-1^∙g^-1^) and wax composition (µg∙dm^-2^). Pearson’s correlation coefficients (*r*) are presented in the upper right triangle, while the corresponding *P*-values for the significance of associations (α = 0.05) are displayed below the diagonal. The different wax classes are as follows: primary alcohol (PA), fatty acid (FA), hydrocarbon (HC), aldehyde (AD), wax ester (WE), and alicyclic (AC). Waxes of the same class are highlighted by red rectangles and brackets.

**Table S1.** Limits of detection (LOD) and quantitation (LOQ) of representative standards for the GC-FID systems and method used in this study.

**Table S2.** The lowest raw data values (µg∙dm^-2^) quantified for wax compounds that had zero values within each environment.

**Table S3.** Means, ranges, and variances for untransformed best linear unbiased predictors (BLUPs) of adult maize leaf cuticular waxes (PA: primary alcohol; FA: fatty acid; HC: hydrocarbon; AD: aldehyde; WE: wax ester; AC: alicyclic; µg∙dm^-2^) for 310 maize inbred lines from two environments in San Diego in 2018, and estimated heritabilities on a line-mean basis.

**Table S4.** Best fitted models used to calculate best linear unbiased predictors (BLUPs) for adult maize leaf cuticular waxes (PA: primary alcohol; FA: fatty acid; HC: hydrocarbon; AD: aldehyde; WE: wax ester; AC: alicyclic) according to a likelihood ratio test (α = 0.05). The star (*) indicates that a random effect term was retained in the mixed linear model, whereas the ‘x’ indicates that a fitted random effect term was not significant and removed from the mixed linear model.

**Table S5.** Untransformed best linear unbiased predictors (BLUPs) of adult maize leaf cuticular conductance (*g*_c_; g∙h^-1^∙g^-1^), BLUPs of flowering time (DTA; days to anthesis) and BLUPs of cuticular waxes (PA: primary alcohol; FA: fatty acid; HC: hydrocarbon; AD: aldehyde; WE: wax ester; AC: alicyclic; µg∙dm^-2^) for 310 maize inbred lines from two environments in San Diego in 2018.

**Table S6.** Lambda values used to transform best linear unbiased predictors (BLUPs) of adult maize leaf cuticular waxes (PA: primary alcohol; FA: fatty acid; HC: hydrocarbon; AD: aldehyde; WE: wax ester; AC: alicyclic; µg∙dm^-2^) for 310 maize inbred lines from two environments in San Diego in 2018.

**Table S7.** Transformed best linear unbiased predictors (BLUPs) of adult maize leaf cuticular waxes (PA: primary alcohol; FA: fatty acid; HC: hydrocarbon; AD: aldehyde; WE: wax ester; AC: alicyclic) for 310 maize inbred lines from two environments in San Diego in 2018. Outlier values for each wax trait were replaced by NA.

**Table S8.** Pearson's correlation of *g*_c_ to cuticular wax composition. The upper triangle of the matrix shows Pearson's correlations coefficients between untransformed best linear unbiased predictors for *g*_c_ and adult maize leaf cuticular waxes (PA: primary alcohol; FA: fatty acid; HC: hydrocarbon; AD: aldehyde; WE: wax ester; AC: alicyclic), whereas the lower triangle shows the significance of the correlations as *P*-values.

**Table S9.** Importance scores and ranks of adult maize leaf cuticular waxes (PA: primary alcohol; FA: fatty acid; HC: hydrocarbon; AD: aldehyde; WE: wax ester; AC: alicyclic) from random forest regression to predict cuticular conductance (*g*_c_) in 310 inbred lines of the Wisconsin diversity panel.

**Table S10.** Genomic information (B73 RefGen_v4) and association statistics for the top 0.002% SNPs associated with adult maize leaf cuticular waxes in a genome-wide association study in the Wisconsin diversity panel.

**Table S11.** Genomic information (RefGen_v4) for the candidate genes residing ± 200 kb of the peak SNPs associated with adult maize leaf cuticular waxes in a genome-wide association study in the Wisconsin diversity panel.

**Table S12.** Genomic information (RefGen_v4) for the top 0.25% associated genes for adult maize leaf cuticular waxes in a transcriptome-wide association study in the Wisconsin diversity panel.

**Table S13.** Genomic information (RefGen_v4) for the top 0.25% genes associated with adult maize leaf cuticular waxes in Fisher's combined test in the Wisconsin diversity panel.

**Table S14.** Number of candidate genes identified from a genome-wide association study (GWAS), transcriptome-wide association analysis (TWAS), and Fisher's combined test (FCT) of adult maize leaf cuticular waxes (PA: primary alcohol; FA: fatty acid; HC: hydrocarbon; AD: aldehyde; WE: wax ester; AC: alicyclic).

**Table S15.** Higher confidence candidate genes associated with adult maize leaf cuticular waxes in all three methods (GWAS, TWAS and FCT) and their grouping based on whether they were identified for the same trait or wax class.

**Table S16.** Rice and Arabidopsis homologs of plausible candidate genes identified for adult maize leaf cuticular waxes via a genome-wide association study (GWAS), transcriptome-wide association study (TWAS), Fisher's combined test (FCT), and GWAS hotspot analysis in the maize Wisconsin diversity panel.

**Table S17.** Overlapping candidate genes for maize leaf cuticle biosynthesis and development regulation (Qiao et al. 2020) and adult maize leaf cuticular waxes.

**Table S18.** Genomic hotspots and loci (peak SNPs) associated with adult maize leaf cuticular waxes in a genome-wide association study in the Wisconsin diversity panel.

**Table S19.** Candidate genes associated with adult maize leaf cuticular waxes by GWAS hotspot analysis and their associated traits in TWAS and FCT.

**Table S20.** Overlapping candidate genes for adult maize leaf cuticular conductance (*g*_c_; Lin *et al*. 2022) and cuticular waxes.

**File S1.** Raw data of leaf cuticular wax abundances

**Files S2-11.** SNP marker genotypes used for GWAS

**File S12.** Transcript abundances used for TWAS

**File S13.** Kinship matrix used for both GWAS and TWAS
