# Supplementary figures and images for: Integrative multiomic analysis identifies genes associated with cuticular wax biogenesis in adult maize leaves
Source: G3 (Bethesda). 2024 Oct 10;14(12):jkae241. doi: 10.1093/g3journal/jkae241 (PMC11631437; doi:10.1093/g3journal/jkae241)

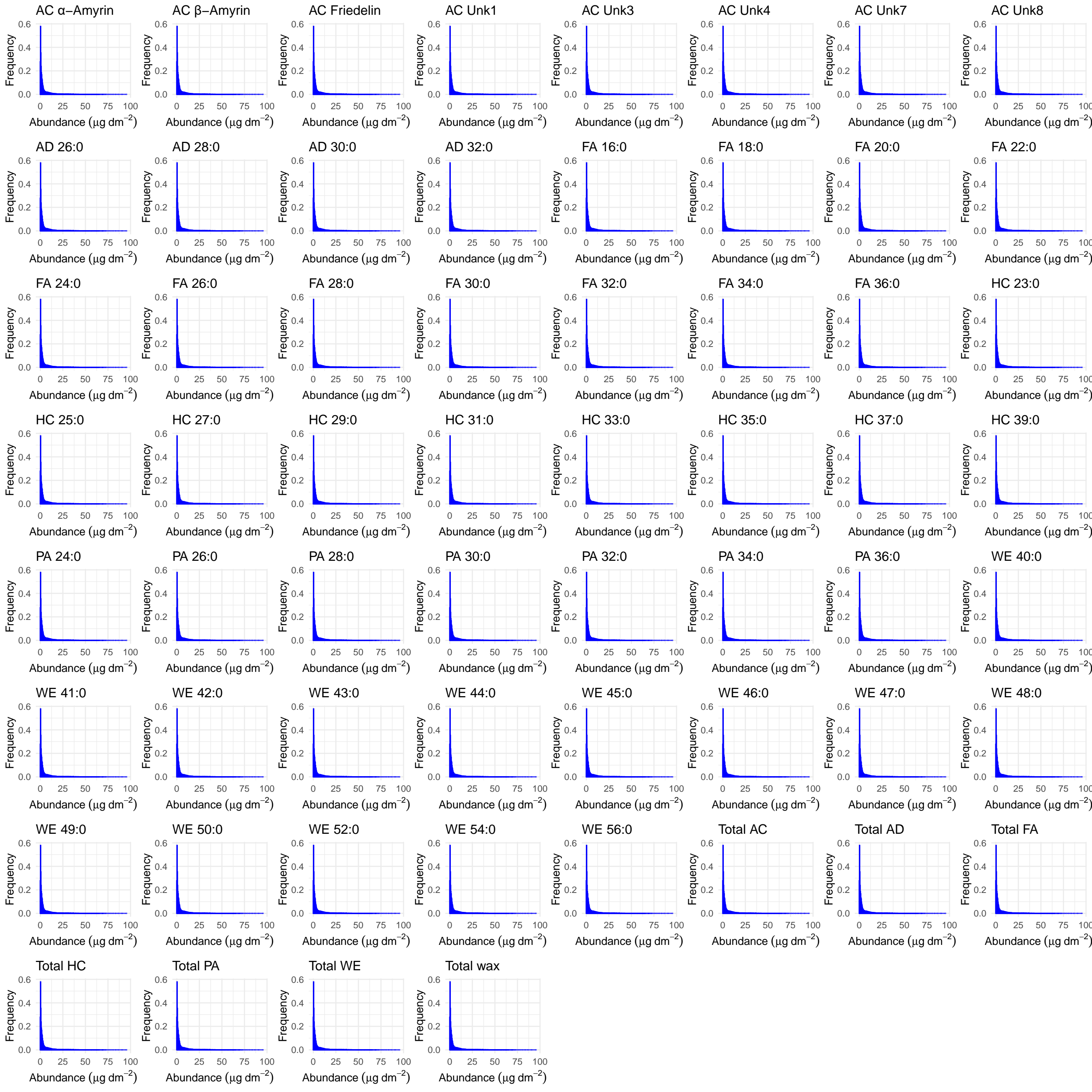

Supplement: jkae241_Supplementary_Data [file jkae241_supplementary_data.zip › Figure_S1_G3-2024-405336.pdf]

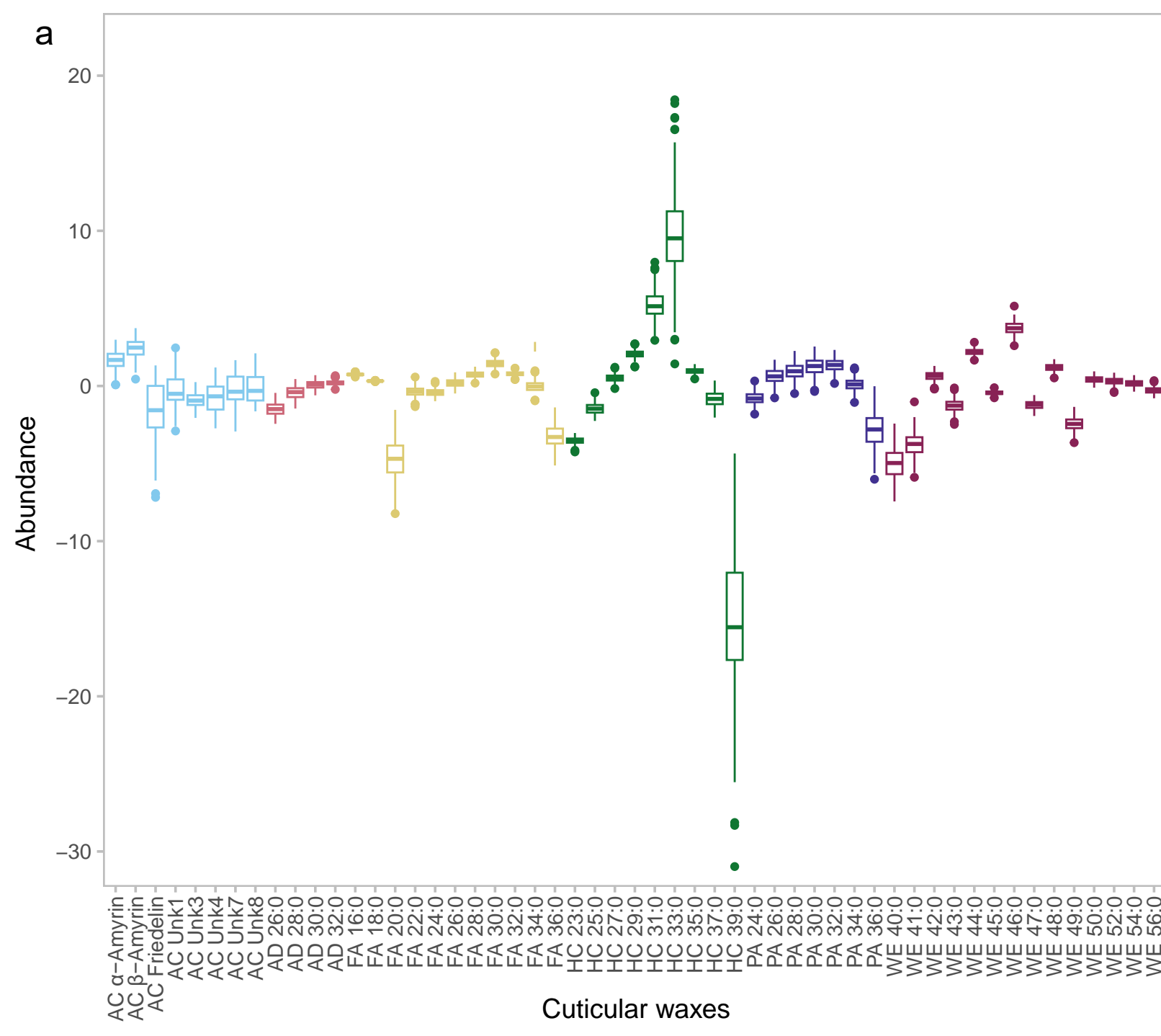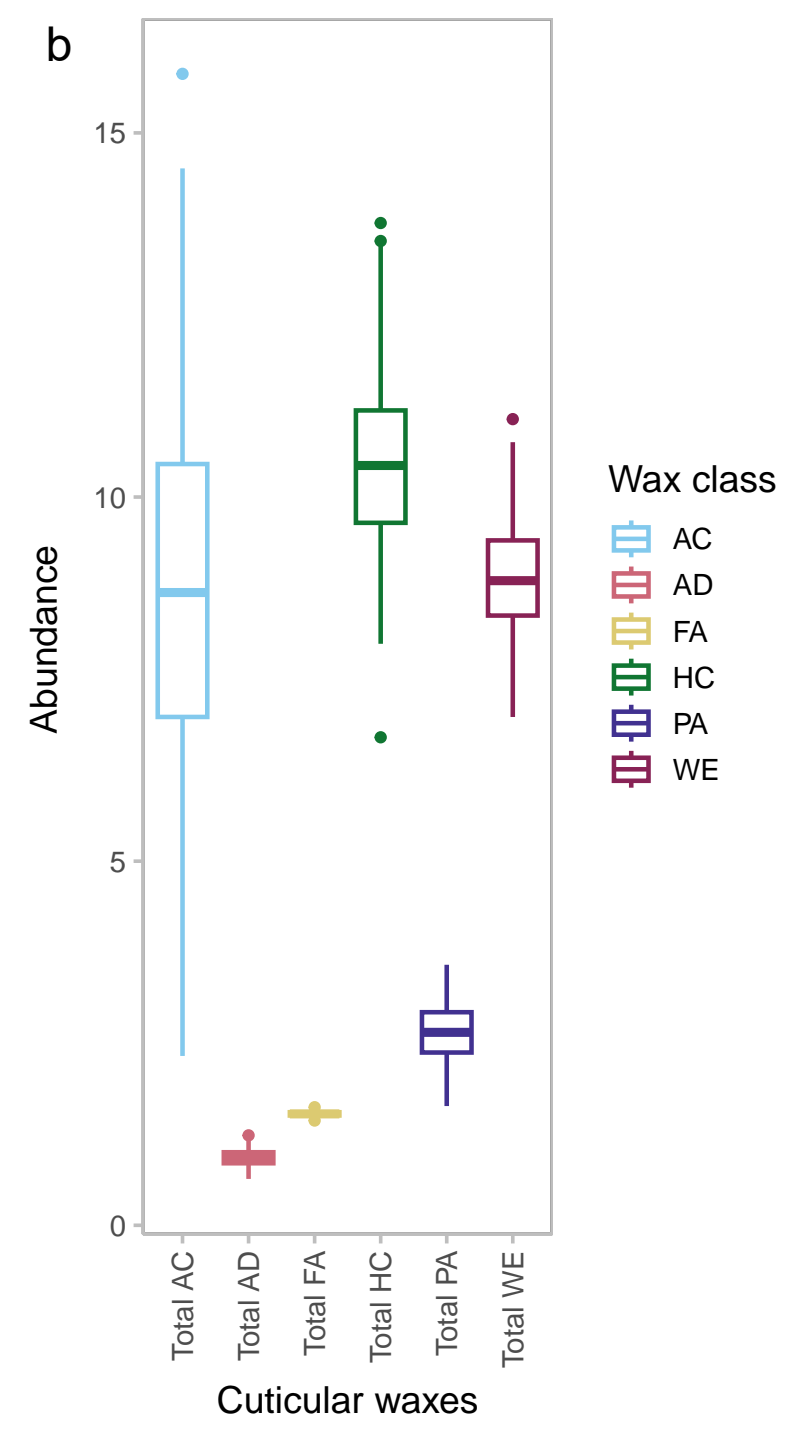

Supplement: jkae241_Supplementary_Data [file jkae241_supplementary_data.zip › Figure_S2_G3-2024-405336.pdf]

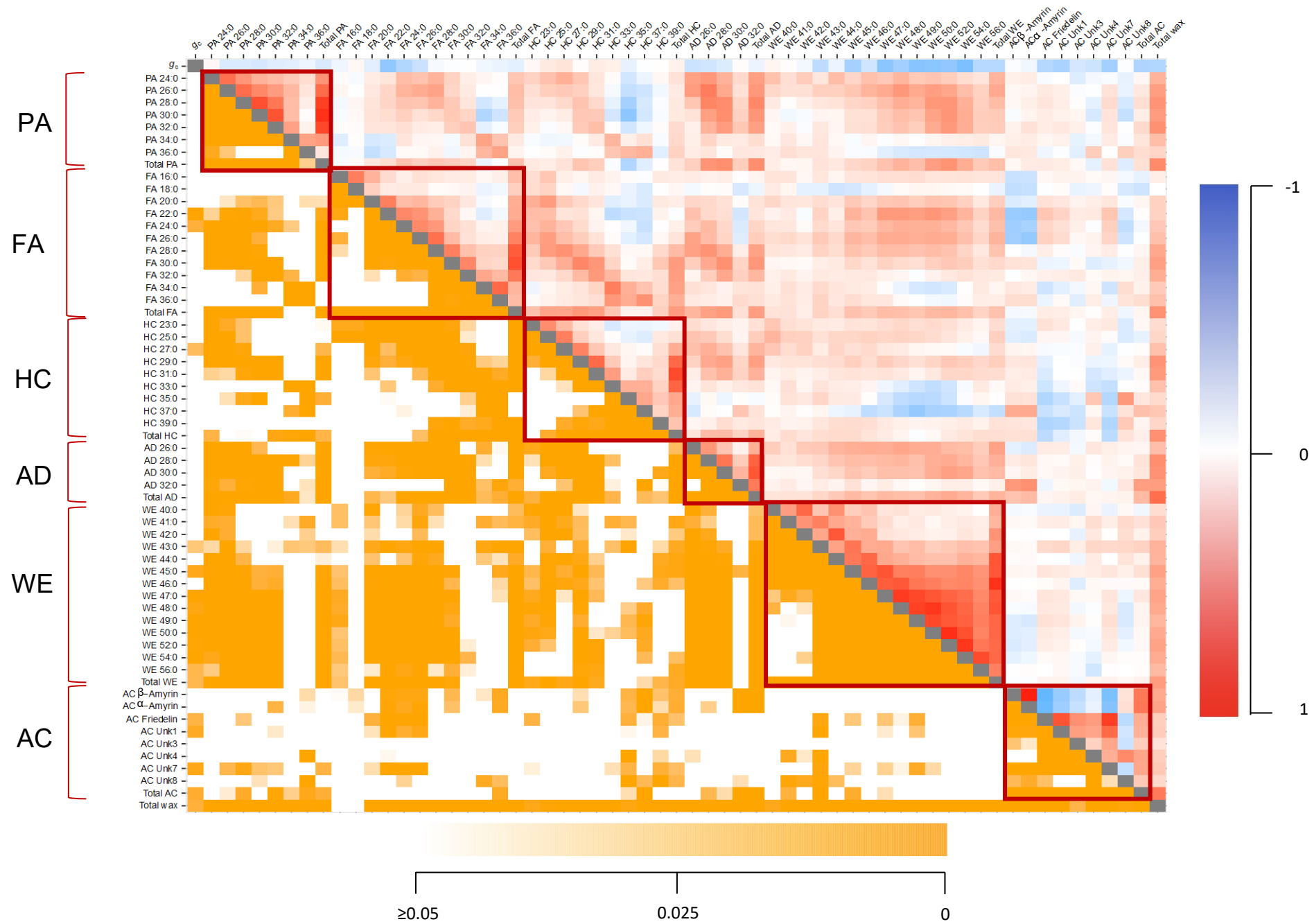

Supplement: jkae241_Supplementary_Data [file jkae241_supplementary_data.zip › Figure_S3_G3-2024-405336.pdf]
